# The Hull Sanatorium for Consumption at Withernsea

**Published:** 1902-10-11

**Authors:** 


### The Institutional Workshop.

### THE HULL SANATORIUM FOR CONSUMPTION AT WITHERNSEA.

Withernsea is about 20 miles from Hull; but such is the miraculous slowness of the trains on that line, that an hour or more is consumed on the journey. The Sanatorium is close to the station, and is a sort of off-shoot of the Convalescent Home, which in its turn has some connection with the Hull Infirmary, although it may not really be a part of that institution. Sir James Reckitt is chairman of the committee, and has taken, and still takes, much interest in all that concerns the welfare of the patients in the Convalescent Home and in the Sanatorium.

The Sanatorium is attached by a corridor to the Convalescent Home, an arrangement whereby the same kitchen is available for both institutions, and economy in construction and in management are obtained.

The Sanatorium is built in linear form, and it faces the south. St. Nicholas' Church is close to. The sub-soil is clay, well drained. Although the view from the windows and verandahs is very limited, those patients who are able to walk about have the whole of the grounds of the Convalescent Home at their disposal for purposes of recreation, and the sea shore is close at hand.

The Convalescent Home is much larger than the Sanatorium, and being placed to the eastward of it, the latter building is much sheltered from the cold east wind, and this condition is further helped by an admirable system of movable screens with which the verandahs are provided.

The sanatorium is two stories in height, and consists in all of four dormitories. Two of these contain six beds each, one contains eight beds and the other ten beds. The floors are of terrazzo, and the angles have in all instances been coved. There are no open fireplaces and the warming is effected by means of hot-water radiators. The windows are on the double-hung sash pattern, and have hopper fanlights. The staircases are of stone. The verandahs are really excellent, and like the wards are floored with terrazzo. The bathrooms and closets project from each dormitory, but they are not invariably cut off from the wards by cross-ventilated passages as they ought to have been, and it might be remarked that the cross-ventilation of the dormitories is not always as perfect as it should be; but it must be remembered that the site of the building is somewhat cramped, and that the architect has fairly well taken advantage of its capabilities. Further it should not be forgotten that the cost of the building was most moderate, only about £3,000 for 32 beds.

The patients are chiefly drawn from the classes of teachers, dressmakers, shop girls, clerks, railway officials, and such like; in fact just those very classes which hesitate to accept gratuitous medical aid and yet cannot afford the £3 3s. or £4 4s. charged at more private institutions. Here the payment is only £1 1s. a week; and when this is said it will be at once realised that the sanatorium cannot fail to do an immense amount of good, and that it has met a real want of the neighbourhood. It goes without saying that the number of beds will soon be insufficient for so populous a town as Hull, and we hope that steps will soon be taken to provide another sanatorium, and that a site having more natural advantages will be selected. In the meantime it is certain that Sir James Reckitt and his fellow-workers deserve much credit for their prompt action in providing arrangements whereby the open-air cure can be carried out at moderate cost for a proportion of victims of phthisis in Hull or its neighbourhood.

Like the convalescent home the sanatorium is under charge of Dr. Sproulle, a medical officer who evidently takes much interest in both institutions.

